# Supplementary material for: Profiling the Physical Performance of Young Boxers with Unsupervised Machine Learning: A Cross-Sectional Study
Source: Sports (Basel). 2023 Jul 7;11(7):131. doi: 10.3390/sports11070131 (PMC10384265; doi:10.3390/sports11070131)
Supplement: Supplementary file 1 [file sports-11-00131-s001.zip › sports-2380999-supplementary.pdf]

**Table 1.** Means, standard deviations, and correlations with confidence intervals

| Variable | *M* | *SD* | 1 | 2 | 3 | 4 | 5 | 6 | 7 | 8 | 9 |
|---|---|---|---|---|---|---|---|---|---|---|---|
| 1. Sex | 1.19 | 0.40 | | | | | | | | | |
| 2. Age | 15.28 | 0.91 | -.09<br>[-.22, .04] | | | | | | | | |
| 3. BM | 63.41 | 15.45 | -.19**<br>[-.31, -.06] | .08<br>[-.05, .21] | | | | | | | |
| 4. CMJ Height | 28.19 | 6.80 | -.40**<br>[-.51, -.29] | .18**<br>[.05, .30] | -.35**<br>[-.46, -.23] | | | | | | |
| 5. Handgrip | 33.45 | 7.65 | -.45**<br>[-.55, -.34] | .27**<br>[.15, .39] | .34**<br>[.22, .45] | .37**<br>[.26, .48] | | | | | |
| 6. Punch Force | 56.73 | 18.87 | -.08<br>[-.21, .05] | .13<br>[-.00, .25] | -.10<br>[-.23, .03] | .08<br>[-.05, .21] | .06<br>[-.07, .19] | | | | |
| 7. PF/BM | 0.95 | 0.38 | .04<br>[-.09, .17] | .08<br>[-.05, .21] | -.55**<br>[-.63, -.45] | .23**<br>[.11, .35] | -.13*<br>[-.26, -.00] | .85**<br>[.81, .89] | | | |
| 8. PF/HG | 1.78 | 0.70 | .22**<br>[.10, .34] | -.04<br>[-.17, .09] | -.26**<br>[-.38, -.14] | -.17**<br>[-.30, -.05] | -.51**<br>[-.60, -.41] | .79**<br>[.74, .84] | .79**<br>[.73, .83] | | |
| 9. PF/(BM+HG) | 0.61 | 0.23 | .11<br>[-.02, .23] | .05<br>[-.08, .18] | -.48**<br>[-.58, -.38] | .10<br>[-.03, .23] | -.27**<br>[-.38, -.14] | .88**<br>[.84, .90] | .98**<br>[.97, .98] | .89**<br>[.87, .92] | |
| 10. PV | 8.64 | 2.64 | -.08<br>[-.21, .05] | .07<br>[-.06, .20] | -.09<br>[-.22, .04] | .17*<br>[.04, .29] | .11<br>[-.02, .24] | .11<br>[-.02, .24] | .15*<br>[.02, .27] | .04<br>[-.09, .17] | .12<br>[-.01, .24] |

*Note.* *M* and *SD* are used to represent mean and standard deviation, respectively. Values in square brackets indicate the 95% confidence interval for each correlation. The confidence interval is a plausible range of population correlations that could have caused the sample correlation (Cumming, 2014). * indicates $p < .05$. ** indicates $p < .01$.
